# ASSOCIATED FACTORS AND CLINICAL ASPECTS OF COLECTOMY IN PATIENTS WITH ULCERATIVE COLITIS - EXPERIENCE OF A BRAZILIAN SOUTHEASTERN COHORT

**DOI:** 10.1590/S0004-2803.24612025-096

**Published:** 2026-05-22

**Authors:** Sandro da Costa FERREIRA, Rogério Serafim PARRA, Gleici da Silva de Castro PERDONÁ, Rosamar Eulira Fontes REZENDE, Lílian Rose Otoboni APRILE, Omar FÉRES

**Affiliations:** 1 Universidade de São Paulo, Faculdade de Medicina de Ribeirão Preto, Departamento de Clínica Médica, Divisão de Gastroenterologia, Ribeirão Preto, SP, Brasil.; 2 Faculdade de Medicina de Ribeirão Preto, Universidade de São Paulo, Departamento de Cirurgia e Anatomia, Divisão de Coloproctologia, São Paulo, SP, Brasil.; 3 Universidade de São Paulo, Faculdade de Medicina de Ribeirão Preto, Departamento de Medicina Social, Ribeirão Preto, SP, Brasil.

**Keywords:** Ulcerative colitis, pancolitis, colectomy, surgery, Retocolite ulcerativa, pancolite, colectomia, cirurgia

## Abstract

**Background:**

Despite recent advances in the medical treatment of ulcerative colitis (UC) involving the use of biological agents and small molecules, a proportion of patients with UC still require surgical treatment**.**

**Objective:**

This study evaluated patient characteristics and factors associated with colectomy in patients with UC at a tertiary IBD center.

**Methods:**

This is a retrospective and observational study utilizing the database at our university hospital. The sample comprised 232 patients diagnosed with UC, with a mean age of 46.45±15.00 years, 60.3% of whom were women. The patients were divided into two groups depending on whether or not they had undergone colectomy between January 2001 and December 2018.

**Results:**

Of all 50 (21.6%) patients underwent colectomy. Clinical intractability (40.0%) and acute severe colitis (22%) were the main indications for colectomy. Longer disease duration (*P*=0.035), pancolitis (82.0% vs 6.0%; *P*<0.001) when compared to proctitis, higher endoscopic Mayo sub-score (Mayo 3: 22.0% vs Mayo 1*:* 5.5%; *P*<0.0001), and initial use of biological agents (14.0% vs 1.1%; *P*<0.001) were significantly associated with the occurrence of colectomy. There was no difference in the distribution of cases according to sex, race, smoking, previous use of corticosteroids, and extraintestinal manifestations (*P*>0.05).

**Conclusion:**

Our study shows that a longer disease duration, extensive disease (pancolitis), and higher severity of UC at diagnosis were associated with a poor prognosis, culminating in a higher need for surgical intervention.

## HIGHLIGHTS

• In a tertiary Brazilian cohort, 21.6% of patients with ulcerative colitis underwent colectomy.

• Clinical refractoriness and acute severe colitis were the main indications for surgery.

• Longer disease duration and pancolitis were independently associated with colectomy.

• Higher clinical (partial Mayo) and endoscopic Mayo scores were associated with an increased need for surgical treatment.

## RESUMO

**Contexto:**

Apesar dos recentes avanços no tratamento clínico da Retocolite Ulcerativa (RCU) envolvendo o uso de agentes biológicos e pequenas moléculas, uma proporção de pacientes com RCU ainda necessita de tratamento cirúrgico.

**Objetivo:**

Este estudo teve como objetivo avaliar as características dos pacientes e os fatores associados à colectomia em pacientes com RCU em um centro terciário de DII.

**Métodos:**

Trata-se de um estudo retrospectivo e observacional utilizando o banco de dados do nosso hospital universitário. A amostra foi composta por 232 pacientes com diagnóstico de RCU, com idade média de 46,45±15,00 anos, sendo 60,3% mulheres. Os pacientes foram divididos em dois grupos dependendo de terem sido submetidos ou não à colectomia entre janeiro de 2001 e dezembro de 2018.

**Resultados:**

Do total 50 (21,6%) pacientes foram submetidos à colectomia. A intratabilidade clínica (40,0%) e a colite aguda grave (22%) foram as principais indicações de colectomia. Maior tempo de doença (*P*=0,035), pancolite (82,0% vs 6,0%; *P*<0,001) quando comparada à proctite, maior subescore endoscópico de Mayo (Mayo 3: 22,0% vs Mayo 1: 5,5%; *P*<0,0001) e o uso inicial de agentes biológicos (14,0% vs 1,1%; *P*<0,001) estiveram significativamente associados à ocorrência de colectomia. Não houve diferença na distribuição dos casos segundo sexo, raça, tabagismo, uso prévio de corticoide e manifestações extraintestinais (*P*>0,05).

**Conclusão:**

O nosso estudo demonstrou que a maior duração da doença, a doença extensa (pancolite) e a maior gravidade da UC ao diagnóstico estiveram associadas a um mau prognóstico, culminando numa maior necessidade de intervenção cirúrgica.

## INTRODUCTION

Ulcerative colitis (UC) is a chronic immune-mediated inflammatory bowel disease (IBD) of the colon characterized by relapsing and remitting mucosal inflammation that classically begins in the rectum and extends proximally through the colon in a continuous manner^1^ ^,^ ^2^. The incidence of UC has been rising worldwide, particularly in newly industrialized countries, and it is associated with healthcare and societal costs, lower quality of life, and hospitalizations, especially in moderate to severe activity^1^ ^-^ ^3^. The inflammatory nature of UC, if inadequately treated, can result in continuous intestinal damage with increased risks of hospitalizations, colorectal cancer, and surgeries^4^.

The incidence rate of colectomy for medical refractory UC has declined substantially since 2005, parallel with the increased use of anti-TNF agents^5^. Other studies report that patient-level risks of surgery have decreased significantly over time, with a 5-year cumulative risk of surgery of 7.0% in UC. This decrease is multifactorial and may be related to early diagnosis, changes in approach to the management of UC with targeted use of disease-modifying therapies, early and wider use of biologics with better disease control and treat-to-target strategies in routine clinical practice. In addition, the risk of dysplasia in patients with long-standing UC is decreasing, and with advanced endoscopic imaging and therapeutic modalities, several neoplastic lesions, which previously warranted colectomy, are now being managed endoscopically, which also likely contributes to the lower risk of colectomy in patients with UC^6^.

Despite advances in the medical management of patients with moderate to severe UC, a significant number of patients still need surgical approaches during the course of the disease^7^. Kaplan et al. assessed temporal trends of colectomy rates for UC, stratified by emergent versus elective colectomy indication, and showed that, from 1997 to 2009, the use of purine anti-metabolites increased and elective colectomy rates in UC patients decreased significantly. In contrast, emergent colectomy rates were stable, which may have been due to the rapid progression of disease activity^8^. The need for emergency surgery still exists and is associated with substantial morbidity and mortality. Postoperative complications frequently occur after colectomy for UC, predominantly among elderly patients with comorbidities^9^. Patients who were admitted to the hospital under emergency conditions and did not respond to medical treatment produced worse outcomes following surgery performed 14 or more days after admission. The quality of life after colectomy for UC is generally good, but there are persistent issues that impact multiple domains, including psychological and sexual functioning^10^.

Brazil lacks data on the characteristics of patients operated upon for UC, as well as the reasons and risk factors for surgery and morbidity in the perioperative period. Thus, this study aimed to evaluate the characteristics and factors associated with colectomy in patients with UC in a tertiary hospital in Brazil.

## METHODS

### Study design

We conducted a retrospective and observational record review of patients diagnosed with UC in a tertiary hospital in Brazil. The diagnosis of UC was made based on clinical, endoscopic and histopathological aspects, and classification was performed according to the Montreal Classification^11^.

The inclusion criterion was patients diagnosed with UC between January 2001 and December 2018. The exclusion criteria included patients with other types of colitis (undetermined, ischemic, and infectious) and Crohn’s disease.

This study was conducted according to Resolution 466/12 of the National Health Council of the Brazilian Ministry of Health and was approved by the Research Ethics Committee of the local institution (protocol # 3147/2019). All participants received explanations about the study, and those who agreed to participate signed the informed consent form.

### Data collection

The data were obtained from the electronic medical records of outpatients monitored at University Hospital of the Ribeirão Preto Medical School. The information collected included clinical and demographic data of the patients. Information on patients’ demographic characteristics, such as gender, ethnicity and current age, was analyzed. Clinical information was about extraintestinal manifestations (EIM), smoking and duration of the disease from diagnosis to colectomy. Previous medications (corticosteroids and immunomodulators), prior use of biological agents (infliximab, adalimumab, golimumab or vedolizumab) and extent of disease (proctitis, left colitis or pancolitis), as well as partial Mayo score and endoscopic Mayo sub score at baseline of the study, were evaluated.

### Statistical analysis

Statistical analyses were performed using the statistical software SPSS version 22 (SPSS Inc., Chicago, IL). A descriptive analysis was performed for population characterization. The data are presented as absolute numbers, percentages, and means ± standard deviations (SD). Comparisons between subgroups were performed using a *t*-test for independent samples. To compare groups for independent samples and categorical variables, we used the Chi-squared (χ2) test or Fischer exact test where appropriate. Survival analysis was performed using the Kaplan-Meier analysis based on the log-rank test. In the multivariate model, we used binary logistic regression, and the model included the following variables: gender, ethnicity, smoking status, mean UC diagnosis time, age, clinical disease severity, degree of endoscopic activity, disease extension at the diagnosis according to the Montreal classification, EIM, and initial use of biologics. Statistical significance was set at *P*<0.05.

## RESULTS

### Patient characteristics

Among the 232 patients included in the study, 140 (60.3%) were female. The mean age was 46.45±15.00 years, and the duration of follow-up was 13.60±8.74 (1-44) years. There were 193 white patients (83.2%) and 24 patients (14.2%) with EIM, which was mainly articular (peripheral arthritis, sacroiliitis, and ankylosing spondylitis), but there were also cutaneous cases (erythema nodosum and gangrenous pyoderma) and hepatobiliary cases (primary sclerosing cholangitis). Management at the time of diagnosis included oral mesalazine (28.8%), topical mesalazine (11.3%), or both (56.7%), but 53 (54.9%) patients also required oral corticosteroids. The demographic characteristics of these patients are shown in Table 1.

**TABLE 1:** Baseline demographic and clinical characteristics of 232 patients with Ulcerative Colitis included in the study.

Variable	UC patients (n=232)	
	n	%
Age (Mean ± SD)	46.45±15.00	
**Gender**		
Female	140	60.3
Male	92	39.7
**Skin color**		
White	193	83.2
Mixed race	21	9.1
Black	18	7.7
**Extension of UC†**		
E1	64	27.6
E2	56	24.1
E3	112	48.3
**Smoking**		
Yes	41	17.7
No	191	82.3
**Extraintestinal manifestation†**		
Yes	33	14.2
No	199	85.8
**Colectomy**		
Yes	50	21.6
No	182	78.4

UC: ulcerative colitis. †: the montreal classification of IBD: E1: proctitis, E2: left colitis, E3: pancolitis. SD: standard deviation.

Through follow-up, 50 (21.6%) patients underwent colectomy. The main indications for surgical treatment were the failure of medical treatment in 20 patients (40.0%), followed by acute severe colitis in 11 patients (22.0%), massive hemorrhage in 8 patients (16.0%), toxic megacolon in 5 patients (10.0%), stricture formation in 3 patients (6.0%), and associated dysplasia or cancer in 2 patients (4.0%). Comparisons of the baseline clinical and demographic characteristics between UC patients who underwent colectomy and those who did not are described in Table 2.

**TABLE 2:** Comparison between demographic and clinical characteristics of patients with Ulcerative Colitis in relation to the occurrence of the colectomy.

	Colectomy				
Characteristics and Clinical variables	Present (n=50)		Absent (n=180)		*P*
	n	%	n	%	
**Duration of UC (Mean ± SD)**	14.00 ±8.22		11.45±7.30		**0.035**
**Gender**					**0.700**
Female	29	58.0	111	61.0	
Male	21	42.0	71	39.0	
**Smoking**					**0.610**
Yes	7	14.0	34	18.7	
No	43	86.0	179	81.3	
**Initial use of biologicals**					**<0.001**
Yes	7	14.0	2	1.1	
No	43	86.0	180	98.9	
Partial Mayo Score**					
2-4	15	30.0	138	75.8	
5-7	26	52.0	33	18.1	<0.001
>7	9	18.0	11	6.0	<0.001
**Mayo Endoscopic Subcore#**					
1	16	32.0	124	68.1	
2	23	46.0	48	26.4	**<0.001**
3	11	22.0	10	5.5	**<0.001**
**Extension of UC** ^†^					
E1	3	6.0	61	33.5	
E2	6	12.0	50	27.5	**<0.001**
E3	41	82.0	71	39.0	**<0.001**
**Extraintestinal manifestation** ^†^					**0.960**
Yes	7	14.0	26	14.3	
No	43	86.0	156	85.7	

UC: ulcerative colitis. †: the montreal classification of IBD: extension of UC: E1: proctitis, E2: left colitis, E3: pancolitis. # the mayo endoscopic score: mayo 0: inactive disease; mayo 1: mild activity; mayo 2: moderate activity; Mayo 3: severe activity. **partial Mayo Score: <2: remission; 2-4: mild activity; 5-7: moderate activity; >7: severe activity. Variables are expressed as mean ± SD or n (%).

### Factors associated with colectomy

**FIGURE 1:**
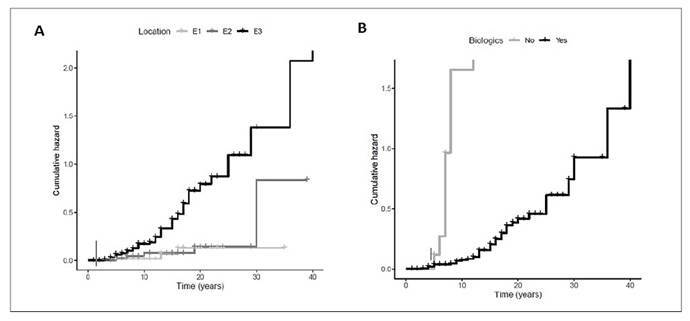
Analysis of the risk of colectomy in UC patients using Kaplan-Meier curves stratified by predictive factors. (A) Cumulative risk of surgical resection was significantly higher in Pancolitis (E3) when compared to proctitis (E1). (B) Cumulative risk of colectomy was significantly higher in UC patients with initial use of biological agents.

**FIGURE 2:**
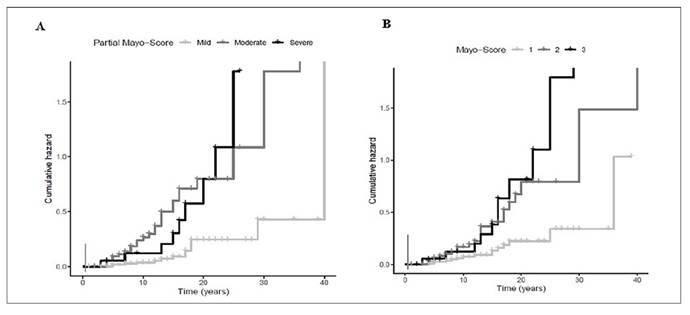
Analysis of the risk of colectomy in UC patients using Kaplan-Meier curves stratified by predictive factors. (A) Cumulative risk of colectomy was associated with higher partial Mayo clinical score. (B) Cumulative risk of of colectomy was associated with higher endoscopic Mayo subscores.

The univariate analysis revealed the following results:

- UC diagnosis time: The mean disease time was significantly longer in the group of patients who underwent colectomy than those without surgical treatment (14.00±8.22 years vs 11.45±7.30 years; *P*=0.035).
- Extension of UC: Pancolitis (E3) was significantly associated with surgical treatment (82.0% vs 6.0%; *P*<0.001; OR=11.74; 95%CI: 3.46-39.82) when compared to those with Proctitis (E1). Figure 1.
- Higher partial Mayo clinical scores were found in patients who underwent colectomy when compared with those without surgical treatment (5-7: 52.0% vs 18.1% and >7:18.0% vs 6.0%; *P*<0.001). Figure 2.
- Higher endoscopic Mayo subscores (Mayo 2: 46.0% vs 26.4% and Mayo 3: 22.0% vs 5.5%; *P*<0.0001) were found in a significantly higher proportion of patients who underwent colectomy relative to controls without surgical treatment. Figure 2.
- The initial use of biological agents was significant in the group of patients who underwent colectomy (14.0% vs 1.1%; *P*<0.001; OR=14.57; 95%CI: 2.92-72.62).
- No associations with colectomy were observed when considering the following baseline characteristics: sex, smoking status, age at diagnosis (<17 years, 17-40 years, >40 years), ethnicity, and presence of EIM (*P*>0.05).

In the multivariate model, we found the following independent risk factors for colectomy: 1) longer disease time (OR: 1.08; 95%CI: 1.04-1.12; *P*=0.005; 2) pancolitis (E3) [OR: 10.23; 95%CI: 2.69-38.83; *P*<0.001]; 3) partial Mayo clinical scores (5-7) [OR: 12.51; 95%CI: 4.85-32.28; *P*<0.001], (>7) [OR: 10.46; 95%CI: 2.92-37.47; *P*<0.001]; 4) use of biological agents [OR: 33.56; 95%CI: 4.42-254.80; *P*<0.001], as shown in Table 3.

**TABLE 3:** Associated factors with colectomy among Ulcerative Colitis patients.

Clinical variables	Univariate analyses			Multivariate analyses		
	OR	95%CI	*P*	OR	95%CI	*P*
Initial use of biologicals	14.57	2.92-72.62	<0.001	33.56	4.42-254.80	<0.001
Longer diagnosis of UC	-	-	0.035	1.08	1.02-1.14	<0.001
Pancolitis (E3)*	11.74	3.46-39.82	<0.001	10.23	2.69-38.83	<0.001
Partial Mayo Score (5-7)**	7.25	3.46-15.20	<0.001	12.51	4.85-32.28	<0.001
Partial Mayo Score (>7)**	7.52	2.69-21.07	<0.001	10.46	2.92-37.47	<0.001

UC: ulcerative colitis. OR: odds ratio. CI: confidence interval. UC: *the montreal classification of IBD: extension of UC: E1: proctitis, E2: left colitis, E3: pancolitis. #the mayo endoscopic score: mayo 0: inactive disease; mayo 1: mild activity; mayo 2: moderate activity; Mayo 3: severe activity. ** partial mayo score: <2: remission; 2-4: mild activity; 5-7: moderate activity; >7: severe activity.

## DISCUSSION

Medical therapy for ulcerative colitis (UC) has undergone significant advances in recent decades, not only due to the use of biological agents, mainly represented by anti-TNF agents (infliximab and adalimumab) and an anti-integrin (vedolizumab)^12^, and more recently a small molecule, the tofacitinib^13^, but also by an improvement in treatment strategies based on well-defined therapeutic targets, such as mucosal healing, in addition to the early initiation of biological therapy^14^. Despite advances in medical management for UC, approximately 20-35% of patients with UC undergo surgery during the course of their disease, with approximately 10% of cumulative risk of undergoing a colectomy in 10 years^15^ ^,^ ^16^.

In this cross-sectional retrospective study, we demonstrated that a significant number of patients with moderate-to-severe UC still need surgical approaches despite the advances in medical management, including the use of biologics.

The surgical treatment of UC is divided into the following settings: urgent, emergent, and elective case^6^. The main indications include acute severe colitis, massive hemorrhage, and toxic megacolon in the context of urgent and emergency surgical treatment, UC refractory to medical management, dysplasia and cancer in the context of elective surgery^17^ ^,^ ^18^.

In our study, 21.6% of UC patients underwent colectomy, and the main indications for surgical treatment were the failure of medical treatment, followed by acute severe colitis and massive hemorrhage.

The long-term course and prognosis of UC are challenging to predict. One of the most extensive population studies evaluating colectomy rates and predictors demonstrated a 10-year cumulative colectomy rate of 8.7%, with residence in northern Europe (compared to southern Europe) and pancolitis being the only predictors of independent associations associated with increased risk of colectomy^19^. More recently, data from the IBSEN study demonstrated that the 20-year cumulative risk of colectomy, after diagnosis of UC, was 13.0%, with pancolitis at diagnosis being independently associated with an increased risk of colectomy when compared with proctitis^20^.

A recent meta-analysis of 14 studies showed that the risk of colectomy in patients diagnosed with UC was 2.8% one year after diagnosis, 7.0% five years after diagnosis, and 9.6% ten years after diagnosis^6^. In our study, longer disease duration and pancolitis were significant predictors of colectomy. A possible explanation for the higher rates of colectomy in patients with longer disease duration could be the presence of dysplasia or cancer, which is probably the leading indication for colectomy in those with long-term UC. Regarding cases of pancolitis, the highest rates of surgery are related to a more aggressive disease phenotype.

Disease activity can directly influence the risk of colectomy in patients with UC, with a correlation between higher disease activity scores and the need for hospitalization for both medical and surgical treatment^21^ ^-^ ^23^. In our series, higher partial Mayo clinical and endoscopic scores were associated with higher colectomy rates in patients with UC, corroborating the results demonstrated in other series^20^ ^,^ ^23^. However, it is essential to emphasize that, despite the availability of several clinical and endoscopic scores to assess and monitor disease activity, these scores may not be routinely verified in clinical practice, especially outside tertiary reference centers, often making it difficult to recognize the patients who might require surgical treatment in the future.

As previously mentioned, considerable advances have occurred in the clinical management of UC over the last two decades, mainly due to the addition of biologics to the UC therapeutic armamentarium, especially tumor necrosis factor-a antagonists (anti-TNF), followed by biological therapies with different mechanisms of action (anti-integrins, anti-IL-12/IL-23), and small-molecule therapies (Janus kinase inhibitors [JAK])^3^. Several population studies and clinical trials in different countries have shown a reduction in colectomy rates in patients with ulcerative colitis in the last two decades^5^ ^,^ ^7^ ^,^ ^8^ ^,^ ^24^. This observed reduction coincides with the introduction of biological therapy in the management of UC^5^ ^,^ ^24^ ^-^ ^26^. However, emergency colectomy rates do not appear to have changed over the said period of time^24^ ^,^ ^26^. Our study observed an inverse association between colectomy and the initial use of biologics. This is similar to the findings of a systematic review that showed no reduction in colectomy rates in UC patients using biologics within South America^27^. One possible explanation is that, in our sample, biological therapy was used in patients with a longer duration of disease and more severe inflammation. These are considered poor predictors of response to treatment and, consequently, a higher risk of colectomy.

Our study provides a comprehensive summary of the factors associated with the occurrence of colectomy in UC patients, but it is not free of limitations. First, this retrospective study included patients followed up at a single reference university hospital. Secondly, our sample size was relatively small. Finally, the studies from tertiary centers are vulnerable to referral and selection bias resulting from a population comprising patients with potentially more serious illnesses.

In conclusion, despite advancements in medical therapy, a significant percentage of patients with UC still need surgical treatment. In our series, the failure of medical treatment followed by acute severe colitis and massive hemorrhage were the most common indications for colectomy. Longer disease duration, extensive disease spread, higher partial Mayo, and higher endoscopic scores at diagnosis were the main factors associated with a higher risk of surgical colectomy.

Despite its limitations, this study has described the experience of a critical IBD reference center in Southeast Brazil. The study provides essential information on the rate and factors associated with colectomy in patients with UC. Strategies such as adequate initial clinical treatment, multidisciplinary approaches, and strict monitoring must be adopted in managing these patients while aiming to identify factors associated with a higher risk of colectomy.

## Data Availability

Data-available-upon-request.
